# Medicines and universal health coverage: challenges and opportunities

**DOI:** 10.1186/s40545-015-0028-4

**Published:** 2015-02-16

**Authors:** Maryam Bigdeli, Richard Laing, Göran Tomson, Zaheer-Ud-Din Babar

**Affiliations:** Alliance for Health Policy and Systems Research, World Health Organization, 20 Avenue Appia, CH-1211 Geneva 27, Switzerland; Boston University School of Public Health, 801 Massachusetts Avenue, Boston, MA 02118 USA; Department of Learning Informatics Management Ethics and Public Health Sciences, Karolinska Institutet, SE-171 77 Stockholm, Sweden; School of Pharmacy, University of Auckland, Private Mail Bag 92019 Auckland, New Zealand

The goal of Universal health coverage (UHC) is to ensure that all people obtain the health services they need without suffering financial hardship when paying for them [1]. This goal is based on the idea of an affordable continuum of care from preventive to curative and rehabilitative services for which essential health system resources are required. These resources include health workers, essential medicines and technologies [1]. Universal health coverage (UHC) is also the focus of ongoing high-level discussions, a momentum created by the World Health Report 2010 and a pathway to which many agencies and governments now commit [2,3].

Essential medicines are considered an integral part of UHC; they are an indispensable element for delivery of services and are also a requirement for high-quality care. It is obvious that careful consideration must be given to ensuring reliable access to quality assured essential medicines when designing benefit packages. Policy instruments that contribute to the effectiveness of the concept of essential medicines include National Essential Medicines Lists, formularies, standard treatment guidelines and measures to ensure access to affordable quality assured medicines.

Aside from being a commodity that is required for service delivery, medicines also contribute significantly to government and household spending on health. Medicines account for over a quarter of total health expenditures with some Low and Middle Income Countries (LMICs) spending up to 67% of their total health expenditures on pharmaceuticals [4]. In low and middle income countries, more than half and sometimes up to 90% of expenditures on medicines are out of pocket [4], putting the most vulnerable households at great risk of financial hardship and catastrophic health expenditures. While part of this spending brings good value for money, medicines contribute to the leading sources of health system inefficiency [3]. This is mainly due to high medicines prices, the variable quality of medicines available on the market (i.e. substandard and falsified medicines) and the inappropriate use of medicines [3].

Some health financing arrangements try to address the issue of high medicines expenditures and medicines waste through cost containment strategies such as limiting access to some medicines categories or putting higher co-payment levels. These measures must be implemented with caution to avoid compromising equity and quality of care [5,6]. Universal Health Coverage has however the leverage to implement sound medicines management strategies that would ensure an effective use of health budgets while maintaining the objective of equitable access to quality assured well selected essential medicines used rationally [7]. These strategies range from evidence-based medicines selection; transparent procurement mechanisms and pricing policies; contracting arrangements with prescribers and dispensers; and strategies to achieve appropriate utilization of medicines including within specific disease programmes. A key element of any UHC essential medicines strategy will be the promotion of generic medicines [3]. As demonstrated by Cameron et al. LMICs could consistently save 60-80% of their medicines expenditures on off-patent originator medicines through the use of unbranded (INN) generic equivalents [8].

Universal Health Coverage has also the convening power to bring relevant stakeholders together, including those who are not usually part of health system strengthening debates, for example both innovator and generic medicines manufacturers, procurement officers or community pharmacy retailers. As health systems of LMICs are increasingly pluralistic, engaging multiple stakeholders in successful dialogue is important and medicines represent an issue for which the diversity of stakeholders is particularly challenging. Decision makers in health systems of LMICs must use these opportunities for dialogue, to effectively balance the competing objectives and interests related to medicines in UHC: ensuring availability of quality assured generic and innovative products, improving equity in access, encouraging appropriate use and keeping costs affordable [9].

World Health Report 2013 highlighted the importance of research for UHC [10] in terms of providing sound evidence for decision making; monitoring impact of policies and interventions; and documenting implementation challenges and successful implementation arrangements. How to ensure reliable access to existing pharmaceuticals and promote innovation for medicines to meet existing unmet needs must be included on this research agenda. This is also vital in improving medicines integration in health system strengthening and towards attaining the goal of UHC.

The papers in this theme issue present a valuable contribution to the above mentioned objectives covering current key pharmaceutical issues in health systems of Asia Pacific and other countries.

The paper on risk sharing schemes in Asia-Pacific describes the current experience and future potential of patient access schemes in South Korea, New Zealand and in Australia. The paper notes that Australia has the most experience with patient access schemes and with the proliferation of high-cost medicines; the use of these schemes may increase to address rising cost pressures, consumer demands, and uncertainties, while attempting to provide patient access within finite budgets. Another paper narrates the uptake of new antidiabetics including insulin analogues and thiazolidinediones in Brazil, China, and Thailand. Brazil was the earliest adopter of insulin analogues while sulfonylureas and metformin dominated the markets in China and Thailand.

A paper on the use of oral antidiabetics in Taiwan outlines a strategy to promote refilling prescriptions in community pharmacies. The paper indicates that the strategy may have had some impact on patient refill behavior. A paper on Brazil’s Farmacia’s Popular Program (FPP) analyses the medicine subsidy policy and access to medicines in the country. It was observed that there has been a substantial increase in the number of pharmacies participating in the FPP programme overtime. This has led to greater programme coverage and has potentially improved access to medicines in the country.

These series of papers come in line with recent publications that provide a different, potentially innovative framework for considering medicines in health systems [11,12]. This is a whole new area of work that opens up to both pharmaceutical policy and health policy and system researchers, who can come together and bring their respective expertise to shed light on medicines situations with a system perspective. Health system decision makers should also be part of this future collaborative work, as they are the main users of the outputs. This endeavor offers opportunities to use increasingly available routine data on medicines or apply health systems, health policy and implementation research methods [13,14] to the field of pharmaceuticals. This should be facilitated by existing initiatives such as the Alliance for Health Policy and Systems Research or Health Systems Global, which have the mandate to foster collaboration among researchers, and between researchers and policy makers; to promote innovation and advocate for a more effective use of evidence in policy.
